# Developing a Competency-Based Learning and Assessment System for Residency Training: Analysis Study of User Requirements and Acceptance

**DOI:** 10.2196/15655

**Published:** 2020-04-14

**Authors:** Cheng-Ting Hsiao, Fremen ChihChen Chou, Chih-Cheng Hsieh, Li Chun Chang, Chih-Ming Hsu

**Affiliations:** 1 Department of Emergency Medicine Chang Gung Memorial Hospital Puzi City, Chiayi County Taiwan; 2 School of Medicine Chang Gung University Taoyuan Taiwan; 3 Chang Gung Medical Education Research Centre Taoyuan Taiwan; 4 Center for Faculty Development Department of Emergency Medicine China Medical University Hospital Taichung Taiwan; 5 School of Medicine China Medical University Taichung Taiwan; 6 Department of Emergency Medicine China Medical University Hospital Taichung Taiwan; 7 Management Chang Gung Memorial Hospital Yunlin Taiwan; 8 Nursing Chang Gung University of Science and Technology Taoyuan Taiwan; 9 Medical Education Department Chang Gung Memorial Hospital Puzi City, Chiayi County Taiwan

**Keywords:** competency-based learning, milestones, entrustable professional activities, assessment, learning platform

## Abstract

**Background:**

The increasingly complex medical environment highlights the importance of milestones and entrustable professional activities (EPAs) to realize the ideals of competency-based medical education (CBME). However, if enormous amounts of assessment results need to be compiled, the development of a digital system to manage, integrate, and synthesize learning and assessment data will be necessary. Furthermore, this system should be able to facilitate real-time assessment with feedback and therefore enhance users’ learning through coaching in the moment in the clinical workplace.

**Objective:**

The main purpose of this study was to develop a competency-based electronic platform system to provide resident physicians with clinical assessments and learning in order to enhance the learning of trainees and reduce the burden of assessments.

**Methods:**

A competency-based learning and assessment system (CBLAS) for residency training was designed, developed, and evaluated in this study. Opinion interviews and a focus group consensus meeting of key users, including trainees, clinical teachers, and administrative staff, were conducted as needs assessments. The structure of the CBLAS was designed according to the thematic analysis of needs assessments. Clinical teachers’ acceptance of using CBME assessments, according to the constructs of attitude, perceived usefulness, and perceived ease of use, was surveyed in the beginning and half a year after implementation of the CBLAS. Additionally, the satisfaction of using the CBLAS, according to information, system, and service qualities, was surveyed after implementation.

**Results:**

The main functions of the CBLAS, including milestones, EPAs, learning portfolios, teacher/student feedback, e-books, learning materials, assessment progress tracking, and statistical analysis of assessment results, were designed and developed for responding to nine themes, which emerged from the needs assessments of the three user groups. Twenty clinical teachers responded to the CBME assessment acceptance surveys before and after CBLAS implementation, which revealed a significant improvement in the factor of “attitude” (*P*=.02) but no significant differences in the two factors of “usefulness” (*P*=.09) and “ease of use” (*P*=.58) for CBME assessments. Furthermore, satisfaction surveys were performed in 117 users, and 87.2% (102/117) were satisfied with the CBLAS in terms of information, system, and service qualities. There was no significant difference in satisfaction among different user groups.

**Conclusions:**

The CBLAS is a user-centered platform that supports clinical teachers’ assessment exercises and residents’ learning, as well as administrative work for staff according to users’ needs assessments and operationalized features of CBME assessments. With the system, clinical teachers had a more positive attitude to conduct the assessment activities of milestones and EPAs and learners could arrange their study schedules to enhance their learning effectiveness. The CBLAS sheds light on how to effectively design and develop a digital system to execute milestone- and EPA-based assessments for enhancing competency-based education among residents, according to our experiences in Taiwan.

## Introduction

### Background

Medical education systems require continual improvement if they are to react effectively to the challenges of rapid changes in technology and the sociocultural environment of the 21st century [1]. To address the medicine-related challenges at hand, equipping doctors with essential competencies has been strongly emphasized in competency-based medical education (CBME) [2]. CBME focuses on practice-based competencies, which have been analyzed and developed according to the needs of the society and patients. CBME does not emphasize time-based training. Instead, it promises greater accountability, flexibility, and learner-centeredness [3]. However, to enable implementation of CBME in the usual clinical teaching and training assessment routines, the most influential and persuasive specific practices are those of “entrustable professional activities (EPAs)” proposed by the Dutch scholar ten Cate [4] and “milestones,” which were planned by the Accreditation Council for Graduate Medical Education (ACGME) [5].

### Competency-Based Medical Education

Medical education in the United States and Canada, as reported by Flexner in 1910, spurred a reform movement, which laid the foundation of a structure- and procedure-oriented medical education model [6]. In this previous report, Flexner mentioned that medical students should have a foundation in elementary science before admission to medical schools. In addition, medical schools should provide students with clinical training, laboratory training, full-time teachers, and a teaching hospital. It was not until 2010 that the Carnegie Foundation for the Advancement of Teaching published “Educating Physicians,” which was like the second version of the report by Flexner. This report clearly outlined the following five major plans for medical education reform in the 21st century: (1) standardization of learning outcomes and individualization of the learning process; (2) integration of formal knowledge and clinical experience; (3) establishment of a holistic medicine and health care team; (4) self-learning and growth; and (5) professionalism and recognition. These reform plans resonate with the CBME model.

CBME, proposed by the World Health Organization in the 1970s, aims to educate “health professionals who can practice medicine at a defined level of proficiency, in accord with local conditions, to meet local needs [7].” After the Institute of Medicine published its report “Crossing the Quality Chasm” in 2001, this important educational issue re-emerged and was widely discussed. CBME is seen as a different teaching method, as it predefines the competencies medical students should acquire before graduation [8] and suggests different methods of evaluating educational outcomes [9]. To identify desirable training results, the concept of “progression of competence” was created [10]. As CBME emphasizes learner-centeredness in students, learners enjoy greater autonomy and flexibility in scheduling their curricula. Moreover, the clear learning goals and the transparent process differ from traditional medical training. Thus, this educational model can better meet the needs of both the society and patients [2,11-13].

### Entrustable Professional Activities

An EPA, proposed by ten Cate in 2005 [4], is defined as unsupervised task execution once the learner has attained a sufficient level of competency. During the assessment activity, the clinical teacher should observe and assess the trainee’s performance and give feedback accordingly [14]. The term “entrustable” was coined to emphasize the importance of “trust” and to reinforce an “entrustment decision” as well [15]. Entrustable competencies are an important aspect of the doctor-patient relationship. Patients visit doctors for treatment because they trust them. In addition, when teachers teach students to attain a certain proficiency level, they also “trust” them to be able to practice their skills in a professional task independently [5]. EPAs embody the core competencies that residents must learn. Through assessment activities when performing a task, residents improve their proficiency of relevant competencies in a professional context, which ensures the realization of CBME and advances medical education.

### Milestones

The concept of residency milestones originated with Nasca, the chief executive officer of the ACGME in 2008, according to the guidelines of which the following two points were mentioned: (1) development of milestones for each specialty and (2) development of assessment tools for milestones [16]. Therefore, a milestone implies an achievement or behavior presented by a physician who has the competency to carry out EPAs [17]. Compared with the current residency training program, the competencies emphasized with milestones are closer to the competencies required by a specific specialty. Milestones specify the core knowledge, skills, attitudes, and beliefs a medical specialist should acquire; moreover, it could also be applied to map out the progression of a medical student from admission to graduation.

The concept of milestones to describe a learner’s progress and performance originated from the skill acquisition model proposed by Dreyfus and Dreyfus [18]. Moreover, clinical teachers use milestones as elements of a standardized assessment tool to indicate a learner’s training outcome. The Milestone Project can help achieve the goals set out in the Outcome Project by serving as indicators of the quality of a training project and the progress of a learner. From that, we know that developing core competency–based milestones is a key step in realizing the spirit of CBME [19].

As patients’ conditions vary, assessments based on diverse content, several types of provisions, and multiple dimensions can be helpful to teachers in keeping them well informed of their students’ learning conditions. Furthermore, teachers can use the digital assessment platform to conduct assessments as part of their duty, according to which they can provide useful feedback. They can also simultaneously encourage students to self-reflect and direct them toward revising their learning plans. As a result, students’ competencies can be developed [20].

Berz et al pointed out in their study that many teachers recognized that compared with milestone-based assessment tools, traditional tools are too narrow in scope, while also being inconvenient to use in the continuity of a clinical setting [21]. The development of a new assessment tool could enhance the quality and quantity of the assessment, as well as the satisfaction of users [21].

However, the data produced from assessments required for training programs based on milestones and EPAs are enormous, and compiling such a large amount of data would consume much time and resources. As a result, the implementations of milestones and EPAs have been hindered owing to the restricted medical and administrative resources. Many studies on related literature point out that the digitization of CBME will be critical for its future development. Lockyer et al noted that effective assessment is essential for the implementation of CBME [22]. Further, effective assessment needs effective information management and documentation, as well as continuous improvements to the assessment system [22]. Additionally, Schumacher et al claimed that as effective assessment requires huge amounts of data, highly effective data management is critically important [23]. Therefore, this work needs to be supported by digital assessments and mobile apps.

The collection and management of enormous assessments in the clinical environment need technology support, and the synthesis of data to inform the “progression of competence” of a trainee relies much on information technology. In the United States, according to the requirement of the ACGME, the Clinical Competency Committee is responsible for collecting data on the assessment results of residents every 6 months and holding discussions to decide each resident’s proficiency level and final training outcome [24]. Analysis and discussion to synthesize the data without the assistance of a digital system would be a formidable challenge in many medical environments, such as those in Taiwan, where medical and administrative manpower resources are limited.

For learning whether trainees’ proficiency levels have met the training goals, a digital system is required to achieve the following goals: (1) comprehensively digitize the activities of the residency assessments; (2) allow teachers and students access to the system for online learning and feedback, without time and space restrictions; (3) reduce trainees’ cognitive load and enhance their learning effectiveness; (4) provide trainees clearer learning goals and resources by integrating the assessment activities and learning into one system; (5) support the operation of a huge amount of statistical analyses and diagrams; (6) conduct assessments on the online system; and (7) create a feedback exchange platform where teachers and trainees can freely share their experiences.

Thus far, in Chinese-speaking areas, only few units have conducted a literature review and begun development of CBME digitization. The development of our Competency-Based Learning and Assessment System (CBLAS) is expected to be the pioneer of CBME digitization. Moreover, as the system was developed in Mandarin Chinese, it could serve as an example of system development for other Chinese-speaking countries.

Considering the aforementioned situations and facts, this study set the following three goals: (1) plan a competency-based and learner-centered training program, objective assessment model, and assessment scale for later integration into the system; (2) design the structure, build a web-based system for residency assessments and learning according to user needs analyses (trainees, teachers, and administrative staff), and implement the system in two medical centers and two regional hospitals in Taiwan; and (3) investigate clinical teachers’ acceptance toward CBME assessments and the assessment time before and after implementation of the system and evaluate system satisfaction by different users after implementation.

## Methods

### Study Methods

The methods of this study included four parts. First, the needs assessments of key users, including trainees, clinical teachers, and administrative staff, for designing the CBLAS were conducted through several opinion interviews and a focus group consensus meeting. Second, the structure of the CBLAS was designed and developed according to needs assessment analyses and consensus. Third, clinical teachers’ acceptance (based on the constructs of attitude, perceived usefulness, and perceived ease of use) of using CBME assessments was surveyed in the beginning of the implementation of the CBLAS and the acceptance was followed up with a survey half a year later. Moreover, we sampled five clinical teachers to observe the average time of performing an assessment in the clinical workplace. Fourth, a total of 117 users (three groups including teachers, trainees, and administrative staff) were surveyed for their satisfaction of using the CBLAS, according to information quality, system quality, and service quality.

### Needs Assessments of Key Users for the Competency-Based Learning and Assessment System

The development of the CBLAS was centered on the users’ needs. We collected opinions from clinical teachers, trainees, and administrative staff regarding the difficulties encountered when performing assessments. The challenges included heavy workload with repetitive tasks, assessment items that were often missed out, waste of time and paper resources to print assessments, and difficulties in recovering backend data. Additionally, we invited five clinical teachers, three trainees, and three administrative staff to participate in a user focus group consensus meeting to discuss the possible solutions for CBME assessment challenges with the web-based system. The records of the consensus meeting and previous opinion interviews were further analyzed with a thematic analysis technique, in which the common themes of the users’ needs were identified by grouping ideas with commonality. We aspired to build a fast and easy system to meet teachers’ and trainees’ needs. Through continuous improvements, modifications, and updates, the functions of competency assessment and teaching were expected to be enhanced. As a result, the learning outcomes could eventually be optimized.

### Development of the System Structure

**Figure 1:**
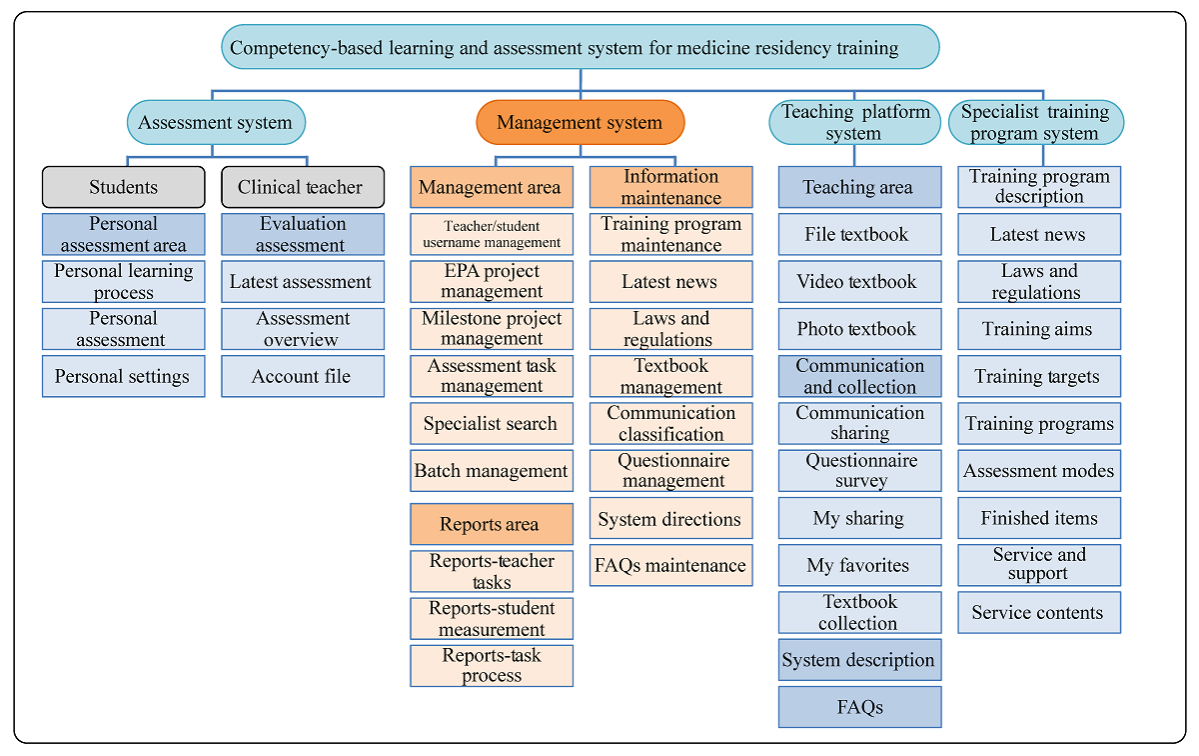
System structure.

Based on the thematic analysis of the users’ needs and the literature review, we divided the CBLAS into the following four major functions: (1) training program, where the training plan, training goals, and program plans of a specific specialty can be viewed and questions regarding the training program can be answered; (2) assessment, where milestones and EPAs are used by teachers and the results can be checked by trainees; (3) learning, where learning materials can be provided and teachers and students can communicate; and (4) management, where the control center of the abovementioned three subsystems is located and is only available to administrators and department secretaries (Figure 1). The details of the design have been reported in the Results with system functions and interface design.

### Acceptance Survey for Competency-Based Medical Education Assessments

The success of CBME relies much on frequent assessments with clinical teachers’ commitment on purposeful observation and meaningful feedback. Clinical teachers’ acceptance of performing CBME assessments would be the key to success. This study applied the constructs in the Technology Acceptance Model, which was proposed by Davis in 1989 [25], to design a survey to investigate clinical teachers’ acceptance of CBME assessments. When users are introduced to a new tool, three main factors influence their acceptance decision [26]. The three factors are perceived usefulness, perceived ease of use, and attitude. Perceived usefulness indicates that users believe that they will improve work performance when using a certain tool. Perceived ease of use indicates that users think that using a particular tool will not need too much effort. The construct of attitude in acceptance represents the users’ emotional perceptions toward using the tool.

**Figure 2:**
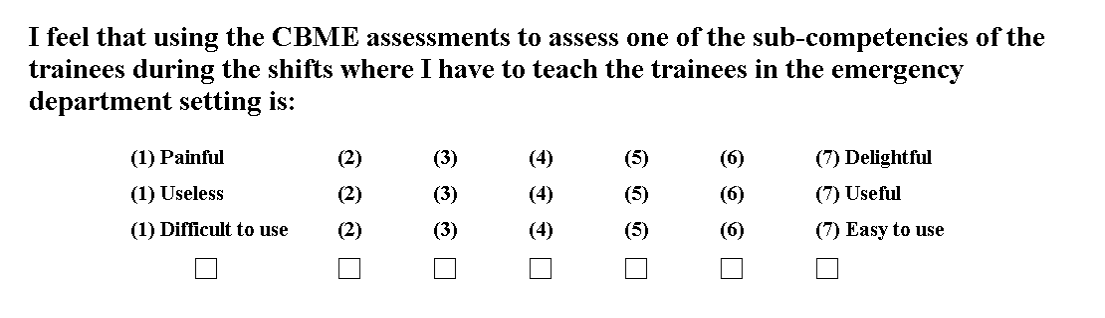
Example item of the questionnaire. CBME: competency-based medical education.

First, we designed the survey with Google Forms to investigate teachers’ acceptance of CBME assessments. The survey questions were designed according to three factors, including attitude toward using CBME assessments (three items), perceived usefulness of CBME assessments (two items), and perceived ease of use of CBME assessments (two items). A 7-point semantic differential scale was used in the questionnaire to rate the variables. An example item of each of the three factors (attitude, usefulness, and ease of use) is shown in Figure 2.

We invited clinical teachers by convenience sampling to respond to the acceptance survey in the beginning of implementation, and they were followed up after 6 months. The paired *t*-test was applied to assess the significance of the difference between the two survey times. We also analyzed the acceptance rate of clinical teachers in terms of each factor, where the percentage of users who rated all items in the factor ≥5 points was considered as the acceptance rate for the factor. Beside clinical teachers’ acceptance, we further sampled five clinical teachers to observe how much time they needed to record their feedback after a clinical assessment with a traditional paper tool and with the CBLAS. The average time for inputting a word (a Chinese character) was counted to descriptively provide a sense of the difference in recording qualitative feedback in the clinical workplace between a traditional paper tool and the CBLAS.

### Satisfaction Survey for the Competency-Based Learning and Assessment System

We assessed users’ satisfaction with the CBLAS after it was implemented for more than half a year. The subjects included clinical teachers, learners, and administrative staff. A questionnaire with a Likert 7-point scale was used in the survey for users to rate their perceived satisfaction in terms of information quality, system quality, and service quality. In the scale, 1 point indicated strongly disagree, 4 points indicated undecided, and 7 points indicated strongly agree with the statement of the question item. Example items of the three factors are as follows: (1) information quality, I feel that the information provided by the CBLAS fits my needs; (2) system quality, I feel that the CBLAS is stable and does not easily crash; (3) service quality, I feel satisfied that CBLAS maintenance personnel can respond to my needs in real time when I encounter problems with the CBLAS.

One-way analysis of variance was applied to analyze the difference in satisfaction between different user groups. When analyzing the satisfaction percentage of the users for a quality factor, we considered scores of all items in the factor >5 points to indicate that users were satisfied with the CBLAS for that factor.

## Results

### Summary of the Needs of Different Key User Groups

After thematic analysis of the records from opinion interviews and a focus group consensus meeting involving five clinical teachers, three trainees, and three administrative staff, we identified the needs that different user groups specified for the CBLAS (Textbox 1).

**Themes of the needs of different key user groups.**

**Residents (trainees)**

1. Learner-centeredness
  - Being able to adjust his/her own learning progress without time and space restrictions
  - Being able to understand and organize future goals through milestones
2. Being able to use different types of devices with the support of responsive web design

**Clinical teachers**

1. Alleviating time- and space-related problems to improve the delivery of training programs and assessment activities
2. Being fully informed of each trainee’s learning progress and needs
3. Being able to provide feedback in real time, communicate, give appropriate assistance, and share knowledge according to the trainee’s competency level

**Medical Education Department (administrative staff)**

1. Reducing the workload in terms of the administrative procedure and large volume of statistical data
2. Reducing paper use and carbon emission
3. Reducing barriers in promoting competency-based training
4. Systematizing records and assessment results

### System Functions and Interface Design

#### Assessment Procedure

The administrator is responsible for creating new personal accounts and managing each batch’s trainees. The department secretaries assign clinical teachers to trainees each month for assessment activities, which include shift-based milestones and EPAs. The CBLAS ensures that a certain amount of assessment results for each trainee comes from different teachers’ assessments so that the reliability and validity of the assessment results can be ensured.

**Figure 3:**
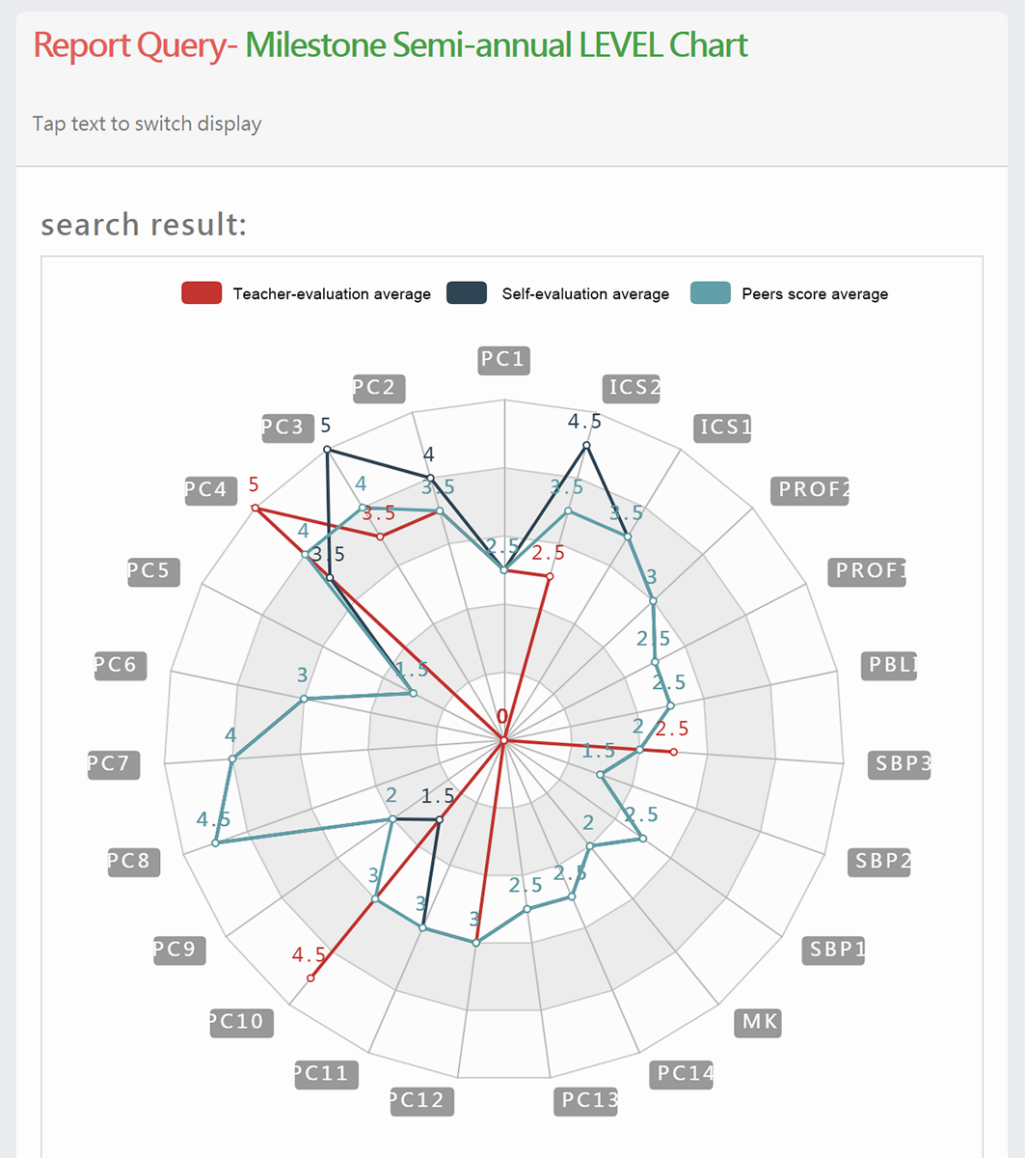
Radar chart.

After data collection, the CBLAS further organizes and analyzes the assessment results. By presenting charts through data visualization, users will become aware of the gap between the expected training goal and the trainee’s actual competency level, which will help with his/her further training. In addition, radar charts are produced to show a trainee’s proficiency level in each subcompetency for indicating whether the trainee is ahead or behind his/her peers (Figure 3). Furthermore, the radar charts serve as a reference for further training required by the learner if the trainee falls behind, so that the goal of teaching each student according to his/her aptitude can be realized.

#### Shift-Based Milestone Interface

**Figure 4:**
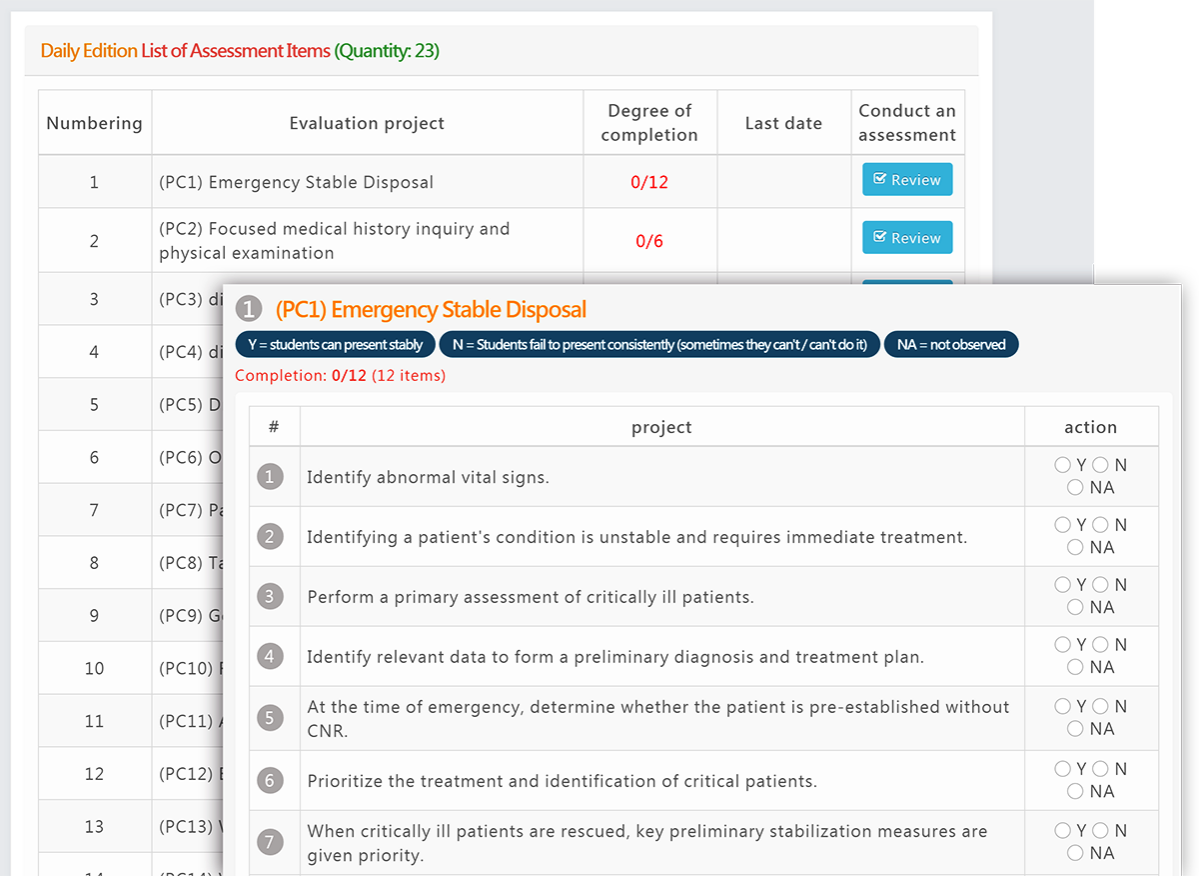
Daily milestone interface.

The shift-based milestone interface is trainee-centered. Teachers assess an assigned trainee’s performance by clicking on the name of the trainee and then on the subcompetency (Figure 4, left) to execute the milestone assessment and feedback activity (Figure 4, right).

#### Half-Year Milestone Interface

**Figure 5:**
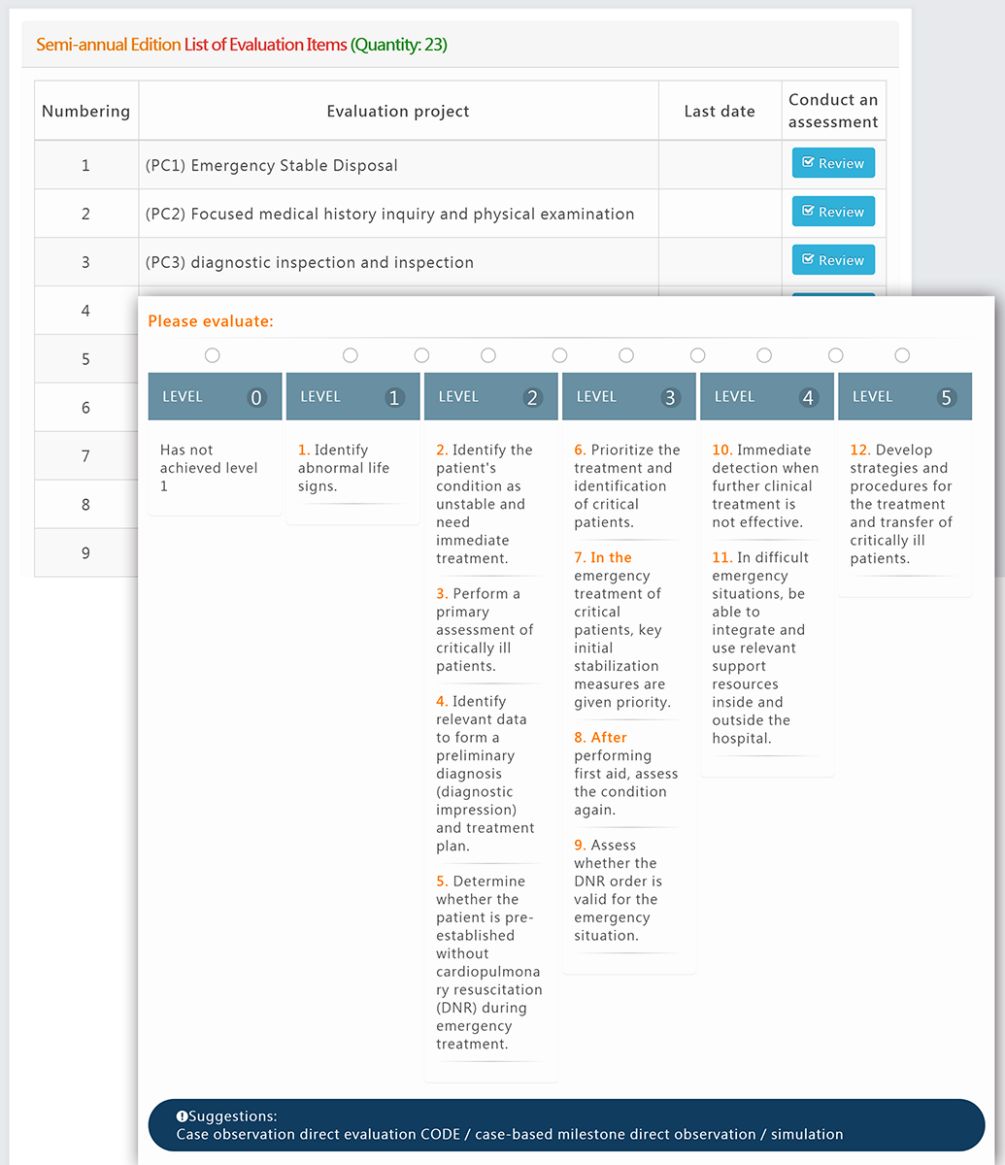
Half-year milestone interface.

The half-year milestone interface is for trainees who have received training for at least 6 months. It is where the assigned teacher provides the assessment to the trainee and the trainee inserts his/her own self-assessment as well. After the teacher clicks on the name of the trainee, the CBLAS shows all 23 subcompetencies to be assessed (Figure 5). The assessment can be saved before the result submission. In other words, the teacher can freely allocate his/her time to complete the assessment activity.

#### Entrustable Professional Activity Assessment Interface

**Figure 6:**
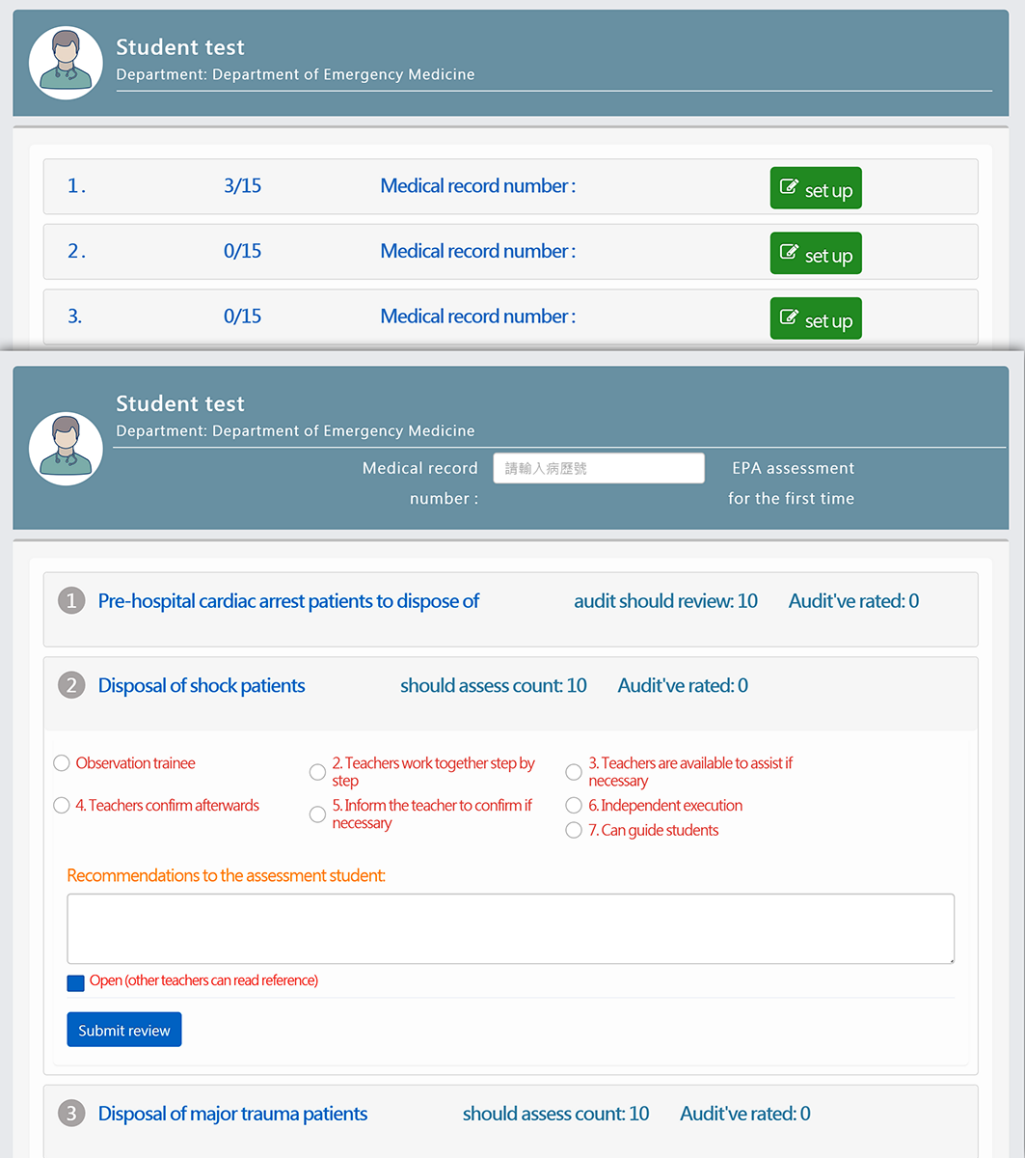
Entrustable professional activity assessment interface.

The EPA assessment interface centers on the trainees and serves as a place where they can communicate with their clinical teachers about the EPAs that need to be assessed. After the clinical teachers click on the name of a trainee and enter the medical number to continue to the next page, they can select the EPAs to provide assessment and feedback (Figure 6).

#### E-Learning Platform Interface

**Figure 7:**
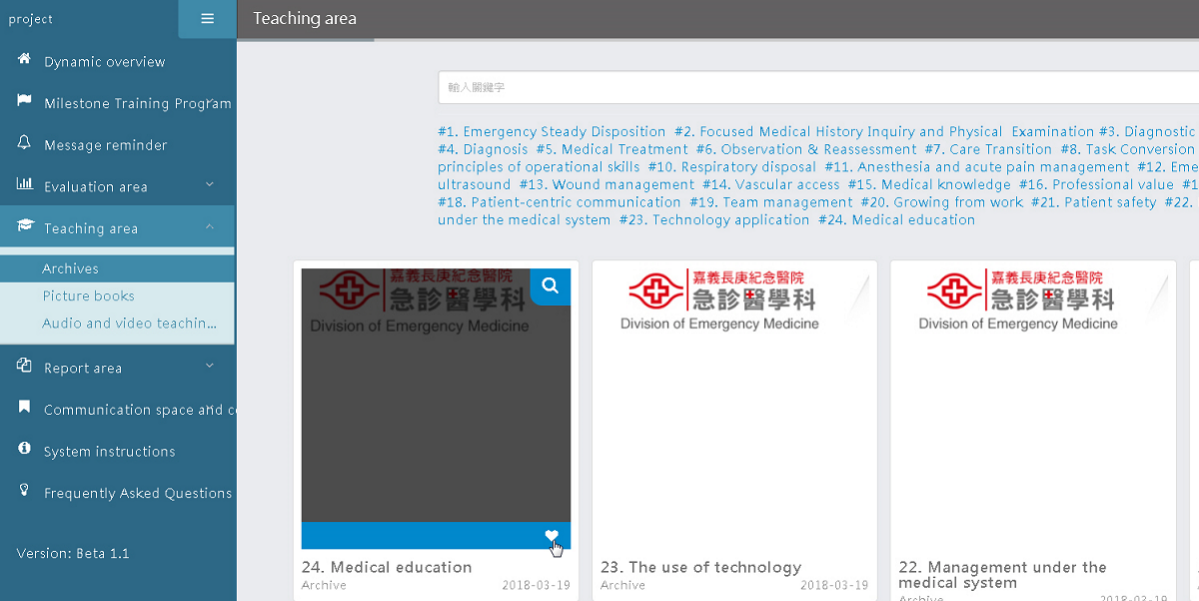
E-learning platform interface.

The e-learning platform interface comprises different specialties, and the learning materials of a specific specialty are shown depending on the trainee’s identity, so that his/her cognitive load can be reduced. Learning materials can be divided into the following three major types: documents, pictures, and videos. Trainees can search for materials based on their needs and can add certain materials into “My Favorites,” so that they can check them at any later time (Figure 7).

#### Instant Notifications and Reminders

Users automatically receive reminders from the CBLAS when they need to provide an assessment. They can simultaneously also receive updates from the CBLAS. Compared with the paper assessments used in the past, clinical teachers have more flexibility in terms of when they need to provide and edit assessments of a trainee’s subcompetencies. No assessment sheets need to be prepared in advance, which can enhance the work efficiency of residency competency assessments. Moreover, the results are shown in the CBLAS in real time, which helps users complete direct observations and clinical assessments at any time and ensures that teachers are fully informed of trainees’ learning progress. As a result, teaching quality and teaching-related work efficiency can be enhanced.

### Clinical Teachers’ Perceived Acceptance of Competency-Based Medical Education Assessments Before and After System Implementation

A total of 20 clinical teachers responded to the CBME acceptance survey before and after the implementation of the CBLAS. We found that their attitudes toward the CBLAS significantly improved after implementation (mean score change from 5.15 [SD 0.72] to 5.70 [SD 0.61], *P*=.02); however, there was not much difference before and after implementation in the perceived usefulness and perceived ease of use for CBME assessments. When we counted the acceptance percentage of the clinical teachers according to their attitude, the acceptance percentage increased from 60% to 90%. The change in the attitude percentage indicated that 30% of the participants changed their attitude from negative or undecided for at least one item in the attitude factor to positive for all items in the attitude factor. The results are presented in Table 1. Beside acceptance, we also randomly sampled five clinical teachers regarding convenience before and after system implementation to observe the time needed for inputting their feedback after an assessment in the emergency department clinical environment. Five different teachers were sampled for the before and after tests. The average time for inputting a word (a Chinese character) reduced from around 2.2 seconds a word by hand writing to 0.5 seconds a word by speech input.

**Table 1:** Acceptance of competency-based medical education assessments before and after system implementation (n=20).

Assessment	Before implementation		After implementation		*t* value
	Score, mean (SD)	Acceptance percentage^a^	Score, mean (SD)	Acceptance percentage^a^	
Attitude	5.15 (0.72)	60%	5.70 (0.61)	90%	−2.55^b^
Usefulness	6.10 (0.91)	90%	6.05 (0.81)	95%	0.191
Ease of use	6.05 (0.89)	90%	5.90 (0.89)	90%	0.557
Overall acceptance	5.77 (0.73)	—^c^	5.88 (0.66)	—	−0.54

^a^Percentage of users who rated all items in the factor with a score ≥5.

^b^*P*<.05.

^c^Three factors were designed to evaluate the acceptance of CBME assessments; therefore, the overall acceptence percentage was not counted in this study.

### Satisfaction Survey for the Competency-Based Learning and Assessment System

A total of 117 users responded to the satisfaction survey, with 52 responses from teachers, 46 from learners, and 19 from administrative staff. The detailed results are shown in Table 2. All three groups of key users showed high scores for the perceived quality of three factors, with the mean score ranging from 5.90 to 6.47 on the 7-point Likert scale. The three factors included information quality, system quality, and service quality. There was no difference in satisfaction among the three groups of users. The satisfaction percentage for all users regarding the three factors ranged from 87% to 90%.

**Table 2:** Perceived quality of the competency-based learning and assessment system (n=117).

Perceived quality	User score, mean (SD)			F (ANOVA^a^)	*P* value	Percentage of satisfaction^b^
	Clinical teachers	Trainees	Administrative staff			
Information quality	5.90 (1.01)	5.98 (1.16)	6.03 (0.62)	1.05	.36	87%
System quality	6.06 (0.75)	6.07 (0.87)	6.15 (0.58)	0.10	.90	90%
Service quality	6.07 (0.83)	6.05 (1.21)	6.47 (0.47)	1.51	.23	89%
Overall	6.01 (0.82)	6.03 (1.02)	6.30 (0.51)	—^c^	—	—

^a^ANOVA: analysis of variance.

^b^Percentage of users who rated all items in the factor with a score ≥5.

^c^Three factors were designed to evaluate the acceptance of CBME assessments; therefore, the overall acceptence percentage was not counted in this study.

## Discussion

### Principal Findings

In the rapidly changing 21st century, the life cycle of knowledge is getting shorter. Lifelong learning will be a normal approach. The CBLAS mentioned in this article is an assessment system for clinical skills learning. It is designed to connect learning with the real workplace to facilitate competency-based performance to a defined proficiency with frequent assessment and feedback to ensure progression of competence [10,14].

Medical education and training have no resistance to rapidly changing technology. Many pedagogical strategies were developed to facilitate an active learner-centered teaching and learning approach [25]. Information technology platforms and assessment tools can provide unique, timely, cost-effective, and valuable opportunities to improve the outcomes of education and training [27]. Heller et al mentioned that the Competence-Based Knowledge Structure for Personalized Learning system focuses on knowledge learning through multifaceted learning to improve learners’ level of knowledge. The assessment takes place when learners finish learning in order to find out whether they have acquired the required knowledge. This e-learning platform emphasizes personalized learning paths and efficient assessment of knowledge and competencies [28]. Zehry et al also mentioned in a 2011 study that these e-learning platforms have several distinct advantages over traditional teaching and learning models, including the ability to update materials in a timely manner for ensuring the ability to provide students with the latest evidence-based content [29]. The CBLAS was designed according to the concept of CBME and the needs of key user groups. This system allows updating of teaching resources, and users can share, search, and discuss on the platform in real time. Thus, it is a valuable resource for updating knowledge. Beyond knowledge and cognition, multiple effective workplace-based assessments and feedback are essential for the implementation of CBME. The CBLAS provides authentic workplace-based assessments and immediate feedback to connect knowledge and clinical practice. We found that clinical teachers’ attitudes had greatly improved after the implementation of the CBLAS. The possible reasons are that the design of this system framework meets the needs of clinical teachers and practical experience promotes the understanding of CBME.

Our study results showed that all three groups of key users had high scores for the perceived quality of the CBLAS. CBME is a paradigm shift from traditional assessments to milestone-based assessments. Schumacher et al found that “Data and Assessment System Development” is an important resource needed to implement this new system [23]. Workplace-based assessments of milestones and EPAs will generate large amounts of data for all trainees across a program. These data will need to be collected accurately and effectively. Additionally, the data should be presented and synthesized in a meaningful manner to facilitate interpretation and review in clinical competency committees. The important point is data accuracy. Every assessment result is only stored in the CBLAS after being confirmed by the clinical teacher. If some assessment items are missed or the same assessment item is checked twice, a pop-up message will remind the teacher to make corrections before clicking on confirm and sending the result back to the CBLAS. The CBLAS is equipped with an automatic submission check function, which can prevent teachers from accidentally failing to assess trainees. Apart from this, the CBLAS matches up teachers with assigned trainees and sends timely reminders to the teachers to avoid repeated assessments and insufficient number of assessments.

In the past, the collection and organization of huge amounts of data were often the most troublesome tasks for administrative staff. The CBLAS can efficiently save time on the delivery of questionnaires because after the assessment is finished, it usually takes few days to submit and collect the paper questionnaire, as well as create a file for it. Further, some teachers even forget to submit the assessment questionnaire. Moreover, the assessment data can be easily categorized, shared, integrated, and analyzed. The CBLAS not only meets the needs of teachers and learners, but also considers the needs of administrative staff.

### Future Plan

Although the CBLAS currently focuses on Western medicine professionals, it has reserved some space to include other specialties in future use. Therefore, we developed an e-learning platform that integrates CBME, which would serve not only as a reference for major departments and countries, but also as a basis for further development. Some improvements were suggested for the CBLAS, and these are presented below.

#### Improve the Program and Learning Content by Simplifying the Content but Targeting the Milestones

To ensure the quality of online learning on different types of devices and to keep pace with the trend of online education, we are consistently making improvements to the CBLAS according to analysis data obtained from pre- and postsurvey user feedback. With a focus on clarifying the learning goals, we made changes to the learning material and teaching content, such as visualizing the content and simplifying the text, which will help users to read the interface on smartphones, tablets, or other devices with ease.

#### Increase the Interactivity to Enhance Community Cooperation and User Engagement

Currently, the teacher-student feedback system has been completed, and in the future, we hope to build a community system where teachers and students can form small learning groups with specific learning goals. Through such a system, teachers and students can have discussions, exchange opinions, and share their learning experiences. As a result, it can increase users’ cooperation in learning and enhance their engagement through smoother group communication.

#### Guide Learners to Access More External Learning Materials and Curricula

Currently, the speed of developing new learning materials cannot keep pace with the speed of medical knowledge advancement. In the future, we hope to find a faster and more effective way of obtaining accurate and appropriate learning materials that teachers can provide to learners in order to ensure that students can easily access learning materials most suitable for them.

### Conclusions

Flexibility and active learning methods take precedence in contemporary medical education [30]. Using an electronic platform for learning will help clinicians avoid geographical and time constraints and promote participation in medical education [31]. Additionally, this will be considered most valuable when using an electronic platform to learn and for its instant feedback, self-assessment, simple interface, extended completion time, and thematic associations [32]. An electronic platform must be taken seriously and adopted quickly, and it should be revised according to the different cultural needs of different countries to facilitate both cognitive knowledge learning and workplace competency-based learning, advocating both learner-centered learning and health care quality [3,7,23,27].

The CBLAS in this study was built with a focus on three key users’ needs to realize competency-based medical education for residency training and to build a visionary learning platform. It not only enhances the quality and efficiency of clinical teachers’ instructions and trainees’ learning progress with milestone assessments at any time, but also addresses administrative heavy workload for educational staff. The CBLAS can show different interfaces according to the user’s identity to fit the user’s needs, which helps reduce the user’s cognitive load and increase the ease and feasibility of CBLAS use. Consequently, the results showed that 87.2% (102/117) of users were satisfied with the CBLAS, without any differences between different user groups.

In conclusion, as expected from the literature [23], our study showed the potential of digital systems to facilitate CBME according to detailed user needs assessments and careful design and development of the system to respond to all the different key users’ needs in the clinical workplace, with operational concepts for CBME, such as EPAs and milestones. More detailed qualitative interviews to explore how the system plays a facilitating role in clinical assessments and the learning process will be the next step to advance our knowledge about the design of competency-based education systems.

## Abbreviations

ACGME: Accreditation Council for Graduate Medical Education
CBLAS: competency-based learning and assessment system
CBME: competency-based medical education
EPA: entrustable professional activity
